# The Release of 24 h Infravesical Obstruction in Mice: Changes in Molecular, Morphological, and Functional Parameters for 14-Day Observation

**DOI:** 10.3389/fmed.2022.892746

**Published:** 2022-05-12

**Authors:** Yutao Lu, Scott R. Manson, Isabela Bastos Binotti Abreu de Araujo, Paul F. Austin, Jens C. Djurhuus, L. Henning Olsen, Rikke Nørregaard

**Affiliations:** ^1^Department of Clinical Medicine, Aarhus University, Aarhus, Denmark; ^2^Department of Urology, Baylor College of Medicine, Houston, TX, United States

**Keywords:** bladder fibrosis, bladder outlet obstruction, animal model, GFR–glomerular filtration rate, release of obstruction

## Abstract

Bladder outlet obstruction (BOO) induces bladder dysfunction and altered bladder architecture. Irrespective of the release of the obstruction, persistent bladder dysfunction severely affects the quality of life. A better understanding of the repair process offers an opportunity to enhance postintervention management. We subsequently evaluated the postobstructive repair process in mice subjected to 24 h BOO followed by release. Male and female mice bladders were obstructed for 24 h by placing a clip around the bladder neck. After the release of obstruction, the mice were studied for 3, 7, and 14 days to observe the bladder repair process over time. Voiding frequency and volume were recorded using the voiding spot assay, and the transcutaneous glomerular filtration rate (tGFR) was measured. Fibrogenesis and associated gene expressions and altered protein levels were evaluated in the bladder using histology, quantatative polymerase chain reaction (qPCR), and Western blot analyses. Bladder wall thickness was increased in both genders over time but occurred later in female mice. Moreover, collagen deposition in the smooth muscle layer increased over time in both genders. Male mice showed a decreased average voided volume at 3 days post release, while female mice showed no significant change during the time course. Fibrosis-related molecular events, including upregulation of fibronectin (FN) protein and Collagen-3 (Col-3) mRNA expression, were transient and normalized again at 14 days in both genders. Transforming growth factor-β (TGF-β) and bone morphogenic protein (BMP)-7 mRNA expressions were upregulated at 14 days post release in both genders. Transcutaneous GFR remained normal during the time course. Release of 24 h BOO initiated a bladder remodeling process. The animal model enables a wide range of experiments to study bladder remodeling, and gender differences offer potential targets for understanding bladder fibrosis and adaptation with BOO.

## Introduction

Bladder fibrosis is characterized by excessive extracellular matrix (ECM) deposition, which is frequently associated with various obstructive conditions, such as benign prostatic hypertrophy, neurogenic bladders, posterior urethral valves, non-neurogenic bladder dysfunction, and urethral strictures. Bladder outlet obstruction (BOO) generates fibrosis including a thickened bladder wall, reduced bladder compliance, changes in innervation, and abnormal muscle contractions (1). BOO activates a series of growth factors, including transforming growth factor-β (TGF-β), promoting the production of ECM, which forms large collagen bundles responsible for poor compliance (2). Targeting TGF-β signaling pathways by knocking out the TGF-β receptor (2) or suppressing TGF-β expression (3) has shown a promising effect in the BOO model by reducing ECM deposition. The effect of TGF-β was further verified in bladder urothelium cells, where silencing SMAD2/3 by siRNA reduced TGF-β-induced epithelial-to-mesenchymal transition (4). Based on these studies, TGF-β has been suggested to play a key role in mediating bladder remodeling.

Although partial BOO in animals is widely used for studying obstructed bladder, the large variation in the degree of obstruction is a significant confounder when results are to be analyzed (5). Previously, we showed that only 24 h total BOO induced bladder architecture alterations and mobilization of factors involved in the fibrosis process with increased fibronectin (FN) expression, upregulation of TGF-β, and phosphorylated SMAD2/3 (6). Surgical removal of obstruction restores urine outflow. However, knowledge about the repair process of the damaged bladder after the removal of the obstruction is lacking. The question is whether this short-term total obstruction can elucidate a repair process biochemically, morphologically, and functionally secondary to release.

Several studies have suggested that the bladder might have a gender-specific response under various pathophysiological conditions, e.g., different responses to medication treatment and different characteristics of bladder cancer (7, 8). The distribution of sex hormone receptors throughout the bladder and urethra in both genders suggests a potential role of sex hormones in bladder pathology (9). We have previously demonstrated gender differences after 24 h of BOO, where increased protein expression of FN and pSMAD2/3 and decreased BMP-7 expression were observed in the male mice but not found in the female mice (6). Whether there is a gender difference following the restoration of urine outflow is unknown.

In this study, we therefore investigated whether a repair process could be identified over 14 days post relief of 24 h of complete BOO and whether gender differences exist for molecular alterations within the bladder wall.

## Materials and Methods

### Animals Model

Experiments were performed using 8-week-old male and female C57BL/6 mice (Janvier Labs, Le Genest-Saint-Isle, France). All mice were housed in a temperature-controlled room with *ad libitum* water and standard rodent chow (Altromin, Lage, Germany) under a 12 h:12 h light-dark cycle at a temperature of 21 ± 2°C and humidity of 55 ± 5%. Mice were acclimatized 1 week prior to the experiment.

In total, 51 male and 51 female mice were randomly allocated into four groups, namely, control (*n* = 13 per sex), BOO was induced for 24 h followed by 3-day release (R-3, *n* = 13 per sex), BOO was induced for 24 h followed by 7-day release (R-7, *n* = 13 per sex), and BOO was induced for 24 h followed by 14-day release (R-14, *n* = 12 per sex). During the surgery, the mice were placed in the supine position on a heating pad to maintain body temperature (37–38°C) under anesthesia induced by inhalation of 3% sevoflurane (Abbott, Scandinavia AB, Solna, Sweden). In the control group, mice bladders were harvested following termination. In the obstruction-release groups, a lower abdominal incision was made to expose the bladder neck, and the proximal urethra was clamped by a vessel clip (1-mm wide and 6-mm long, 15 g pressure; World Precision Instruments, Sarasota, CA, United States). The abdominal wall and skin were closed separately with 6-0 Prolene sutures. The obstruction was maintained for 1 day, and then another surgery was performed to remove the clip and the bladders were emptied by gentle pressing under anesthesia. The mice were returned to the cage and followed up for 3, 7, and 14 days, respectively. At the following-up time points, mice bladder function, histology, and molecular changes were analyzed.

The procedures described were performed in concordance with the Danish national guidelines for animal care and the published guidelines of the National Institutes of Health and approved by the institute’s local committee according to the licenses for use of experimental animals issued by the Animal Experiments Inspectorate, the Danish Ministry of Food, Agriculture and Fisheries (Approval number: 2017-15-0201-01182).

### Void Spot Assay

The method of void spot assay (VSA) was similar to the study by Yu et al. (10). The mice were placed directly on a precut filter paper (30 cm diameter; catalog no. 1,540–320, Whatman grade 540) on top of a wire mesh in a metabolic cage (21 cm in diameter), with free access to food and water. As previously shown, a 4 h VSA caused marked void spot overlapping (11), which was in accordance with our pilot study, therefore changing of paper after 2 h acclimation reduced the void spot overlapping. Thus, 2 h acclimatization prior to the 2 h VSA test was adopted. Testing was performed during a fixed time window (10 am–2 pm) for 4 h.

The R-14 group was investigated at all time points and was therefore used to show the development in the voiding function. The rest of the mice served as control of how representative the study group was at given time points.

After urine had dried, the filter papers were imaged using the ChemiDoc image system (BioRad, Hercules, CA, United States). Images were captured using a fluorescein filter. Exposure settings were optimized to maximize signal over noise. After being electronically captured, the images of the filter papers were quantified for spot counts and areas by ImageJ software (U.S. National Institutes of Health, Bethesda, MD, United States). An area-to-volume standardized curve was developed based on the controlled delivery of urine in different volumes (i.e., 1, 2, 5, 10, 20, 30, 40, 50, 100, 150, and 200 μl) on filter paper in duplicate.

Using ImageJ, the digital images were converted to stacks with removed scale. The “Yen auto-threshold” function was applied to highlight the void spots. The “analyze particles” function was then used to read the spots number and spots area and spots with area <1,500 pixels were excluded to drop those small spots, which were corresponding to 1 ul and could be due to the claws or tails movement.

### Non-invasive Transcutaneous Assessment of Glomerular Filtration Rate

Transcutaneous assessment of GFR was previously established in mice while awake and free to move (12). In short, under light anesthesia (2% Sevoflurane), part of the back region of the mice was depilated. A transdermal reader-sensor device (MediBeacon, Mannheim, Germany) was attached to the skin in the back region using a double-sided adhesive patch (MediBeacon, Mannheim, Germany). Fluorescein-labeled-sinistrin tracer (FITC-S–Fresenius Kabi, Austria), 7.5 mg/100 g body weight, was administrated intravenously (retro-orbital plexus). Mice were kept in separate cages for 1 h. Individual transcutaneous glomerular filtration rate (tGFR) values were given by the elimination kinetics curve of FITC-S. Data were analyzed under 3 compartments using MB Studio (MediBeacon, Germany) (11). All animals were submitted to repeated measurements in different time points but using always the same sensor set (13).

### Histology

Bladders were fixed in 4% paraformaldehyde for 1 h, rinsed with PBS, dehydrated in a series of graded alcohol, and embedded in paraffin. Tissue sections (5 μm sections) were stained with hematoxylin and eosin (H&E) to evaluate bladder wall thickness, and Sirius Red staining was performed to visualize collagen deposition.

The bladder wall thickness was measured at 5 random locations to calculate the average thickness. Images of 5 randomly chosen areas from Sirius Red staining at 10 × magnification were taken using the Olympus cellSens Imaging software (Olympus Corporation, Tokyo, Japan). By using bright-field microscopy with or without polarization filter, the collagen fibers stained by Sirius Red appeared bright red (i.e., collagen I) and bright green (i.e., collagen III) in sharp contrast with the rest of the tissue remaining black. The collagen deposition in the smooth muscle layer was quantified by Image J at 20 × magnification from 5 randomly captured images. The collagen area was presented as the percentage of total tissue area.

### Protein Isolation and Western Blotting

The bladder tissues were divided sagittally into two halves. One half was used for Western blotting and the other part for quantatative polymerase chain reaction (qPCR) analysis. For Western blotting analysis, the bladder tissue was homogenized in radioimmunoprecipitation assay (RIPA) buffer (Thermo Fisher Scientific, Waltham, United States). Protease inhibitors (phosphatase inhibitor cocktails 2 and 3, Sigma-Aldrich, St. Louis, United States) and mini protease inhibitor cocktail tablets (Complete Mini, Roche, Hvidovre, Denmark) were premixed and added to the tissue samples according to the manufacturer’s instructions. A TissueLyser LT (Qiagen, Hilden, Germany) homogenized samples for 4 min at 50 Hz, before centrifugation at 1,000 × *g* for 10 min at 4°C. The samples for gel loading were prepared by adding Laemmli sample buffer containing 2% sodium dodecyl sulfate (SDS) to the supernatant. The total protein concentration was determined using a Pierce BCA protein assay kit (Roche, Hvidovre, Denmark) according to the manufacturer’s instructions. Following electrophoresis separation on 12% Criterion TGX precast gel, sample proteins were electrotransferred to a nitrocellulose membrane. The blots were blocked with 5% fat-free dry milk in PBS-T (14). The blots were then washed with phosphate-buffered saline, 0.1% Tween^®^ 20 (PBS-T) and incubated with primary antibodies overnight at 4°C. Proteins were visualized using an Amersham ECL Plus enhanced chemiluminescence system (GE Healthcare, Chicago, United States) after incubation with horseradish peroxidase (HRP)-conjugated secondary antibodies for 60 min at room temperature. All Western blots were normalized to total protein content, as measured using stain-free technology (15). Primary antibodies included FN (ab2413 Abcam, Cambridge, United Kingdom), Smad1 (6944 Cell Signaling Technology, MA, United States), Smad 2/3 (3102 Cell Signaling Technology, MA, United States), phosphorylated Smad1/5/9 (13820 Cell Signaling Technology, MA, United States), and phosphorylated Smad2/3 (8828 Cell Signaling Technology, MA, United States). Secondary antibody included goat antirabbit immunoglobulins/HRP (P0448, DAKO, Glostrup, Denmark).

### RNA Extraction and Quantitative PCR

Total RNA from the other half of the bladder were isolated using a Nucleospin RNA II mini kit, as stated in the manufacturer’s manual (Macherey Nagel, Düren, Germany). The RNA concentration was measured spectrophotometrically at 260 nm, and samples were then stored at -80°C. The AffinityScript qPCR cDNA synthesis kit (Life Technologies, Thermo Fisher Scientific, Cambridge, MA) was used to synthesize cDNA. For qPCR analysis, 100 ng of cDNA served as the template for PCR amplification using SYBR^^®^^ Green qPCR Master Mix according to the manufacturer’s instructions (Life Technologies) on an Aria Mx3000P qPCR System (Agilent Technologies, Santa Clara, CA, United States) with β-actin as the control gene. The primer sequences for gene of interest are shown in Table 1.

**TABLE 1:** Primer sequences for qPCR analysis of mouse samples.

Primer	Sequence
Mouse TGF-β	
Forward	5′-ACCGGAGAGCCCTGGATAC-3′
Reverse	5′-AGGGTCCCAGACAGAAGTTG-3′
Mouse BMP-7	
Forward	5′-TACGTCAGCTTCCGAGACCT-3′
Reverse	5′-TGGGTTGATGAAGTGAACCA-3′
Mouse Col-1	
Forward	5′-CACCCTCAAGAGCCTGAGTC-3′
Reverse	5′-ACTCTCCGCTCTTCCAGTCA-3′
Mouse Col-3	
Forward	5′- GCACAGCAGTCCAACGTAGA-3′
Reverse	5′- TCTCCAAATGGGATCTCTGG-3′
Mouse β-actin	
Forward	5′- TGTTACCAACTGGGACGACA -3′
Reverse	5′- CTGGGTCATCTTTTCACGGT -3′

### Statistical Analysis

Prism 8.0 (GraphPad Software, Inc. San Diego, CA, United States) was used for statistical analyses. Data have been presented as means ± SD. Multiple comparisons between control, R-3, R-7, and R-14 within gender were performed using a one-way analysis of variance (ANOVA) followed by the Tukey’s multiple comparison test. The unpaired *t*-test was used to compare difference between genders. Pearson correlation was used to calculate correlation between bladder wall thickness and collagen deposition. *P* < 0.05 was considered statistically significant.

## Results

During the experiment, 2 male mice in the R-7 group and 4 female mice (i.e., 2 in R-3 and 2 in the R-7 group) were terminated when removing the 24 h BOO clips due to bladder rupture, and another 2 female mice in R-14 were terminated due to technical problems during tGFR measurement. Organs were harvested from 49 male and 45 female mice, including 13 male and 13 female mice in the control group, 13 male and 11 female mice in the R-3 group, 11 male and 11 female mice in the R-7 group, and 12 male and 10 female mice in the R-14 group. The mice body weights are shown in Table 2 and showed no change over time.

**TABLE 2:** Animals body weight (g).

	Control	R-3	R-7	R-14
Male	24.0 ± 1.8	25.2 ± 1.6	24.3 ± 1.2	24.1 ± 1.2
Female	19.7 ± 2.0	19.2 ± 1.0	19.15 ± 1.5	20.1 ± 2.3

### Assessment of Bladder Wall Thickness and Collagen Deposition

To determine whether a 24 h total infravesical obstruction followed by release induces changes in the bladder morphology, we evaluated bladder wall thickness and performed Sirius Red staining to analyze collagen deposition. The obstruction and following release were associated with significant bladder wall thickness changes.

**FIGURE 1:**
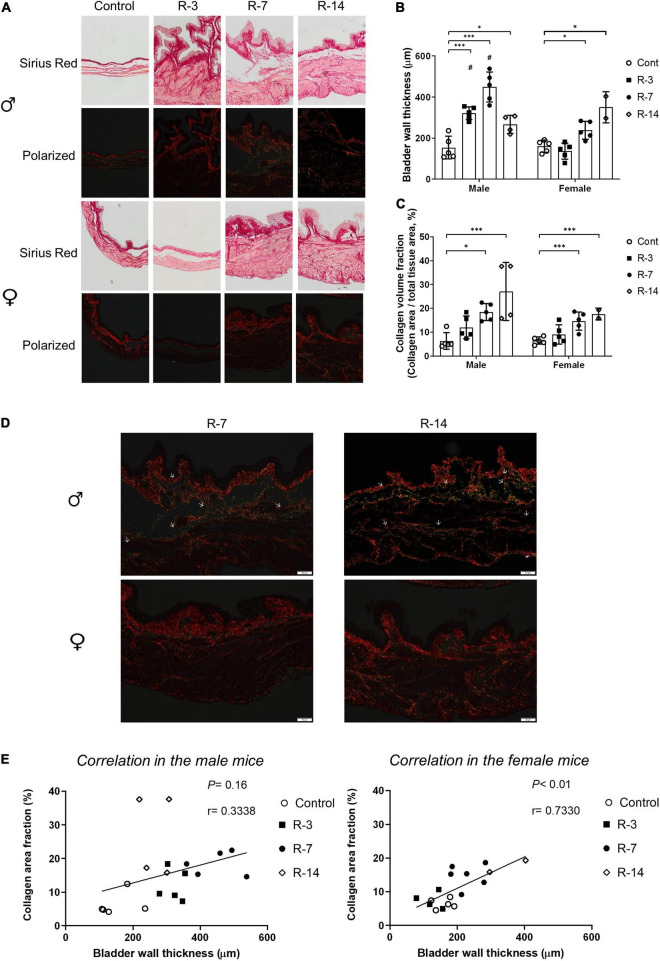
Bladder wall changes after release of 24 h total bladder outlet obstruction detected by Sirius Red staining. **(A)** It illustrated the collagen deposition in the bladder wall. By way of polarized filter, the collagen fiber was bright red/green on the dark background (*n* = 4 in the male R-14 group, *n* = 2 in the female R-14 group, and *n* = 5 in other groups). **(B)** Increased bladder wall thickness was shown in male mice at 3, 7, and 14 days post release, but only 7 and 14 days post release in female mice. The bladder walls in the R-3 and R-7 groups in the male mice were thicker compared with those in the female mice. **(C)** Increased collagen area fraction in the smooth muscle layer was shown in the R-7 and R-14 groups in both genders. **(D)** Deposition of collagen I (indicated in red) was pronounced in the suburothelium and smooth muscle layers in both genders. Collagen III deposition (indicated in green) was more pronounced in the smooth muscle layer in the male 7 and 14 days post release. There was nearly no collagen III deposition in the female 7 or 14 days post release (original magnifications 10×). Arrows show localization of collagen III. **(E)** The increased collagen area fraction did not correlate with the bladder wall thickness in the male mice, while significant correlation was shown in the female mice. Male, ♀ Female. Data are presented as means ± SD. *P < 0.05 and ^***^P < 0.01. # *P* < 0.05 compared between genders with the corresponding group.

In the male mice, the bladder wall thickness was increased at 3, 7, and 14 days post release compared with control mice (Figures 1A,B). In the female mice, an increased thickening bladder wall was delayed and observed at 7 and 14 days post release. There was no change in thickness at 3 days post release (Figures 1A,B). The male mice showed increased bladder wall thickness at 3 and 7 days post release compared with that of females (Figure 1B).

In the control group of both genders, the collagen was largely located in the suburothelial layer, while there was minimal collagen in the smooth muscle layer (Figure 1A). The collagen deposition in the smooth muscle layer increased at 7 and 14 days post release in both the male and the female mice compared with the control group, but no change was observed between the genders (Figure 1C). Additionally, more pronounced collagen I (red color) staining was observed in the smooth muscle layer after the release of obstruction in both the male and female mice compared with the control. In the male mice, evident collagen III (green color) emerged between the smooth muscle layer at 7 and 14 days post release. However, in the female mice, the staining of collagen III was not obvious through the time course (Figure 1D).

There was significant correlation between bladder wall thickness and collagen deposition in the female mice, while no correlation was found in the male mice (Figure 1E).

Taken together, these results indicate that the bladder wall thickness was increased earlier in male mice compared with female mice in response to the release of 24 h BOO, while the collagen deposition increased in both genders at 7- and 14 days post release. The distribution of collagen, including collagen III, was more pronounced in the smooth muscle layers after the release of 24 h BOO in the male mice, and collagen III appears to be preferentially expressed in bladders of male mice.

### Analysis of Voiding Spot Assay and Voiding Function Changes During the 14-Day Observation

**FIGURE 2:**
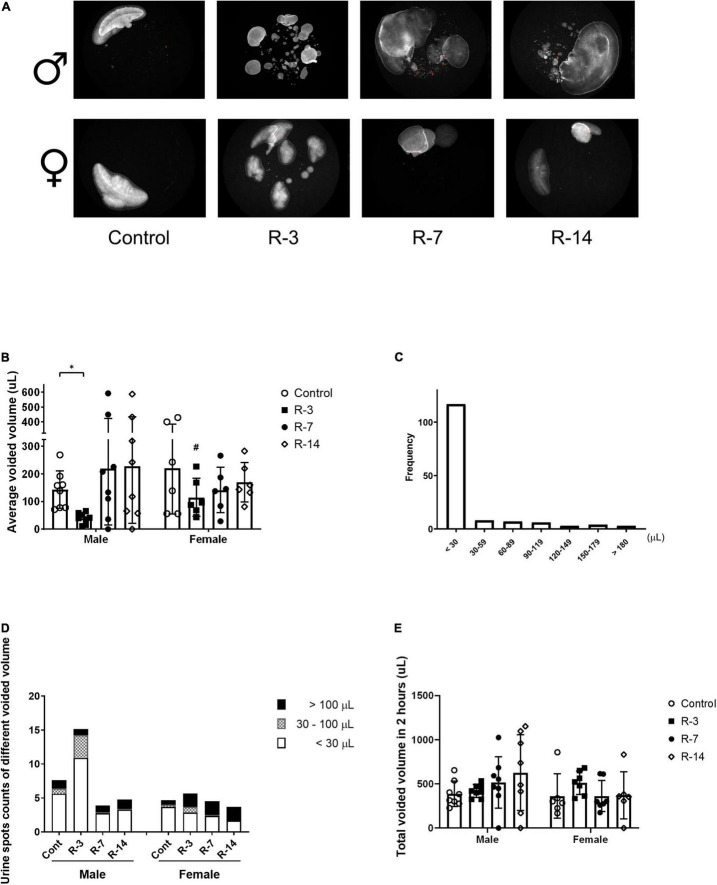
Voiding function changes after release of 24 h total bladder outlet obstruction monitored by voiding spot assay. **(A)** A representative 2 h voiding spot measure (*n* = 8 in each group in the male mice and *n* = 6 in each group in the female mice). **(B)** The average voided volume was significantly decreased in the male mice at 3 days post release and returned at 7 and 14 days to the control levels. The average voided volume in the male R-3 group was smaller than that in the female R-3 group. **(C)** The urine spot distribution by single voided volume in the control mice, of which urine volume less than 30 μl consisted of the majority. **(D)** The urine spots count as in less than 30 μl, between 30 and 100 μl, larger than 100 μl were drawn in the chart. The male mice at 3 days post release showed higher counts than the control, which was mainly due to the increase of small volume voids. The female mice showed no changes. **(E)** The total spot area, represented the total voiding volume in the 2 h recording, showed no changes in both genders during the observation period. Male, ♀ Female. Data are presented as means ± SD. *P < 0.05. # *P* < 0.05 compared between genders with the corresponding group.

To evaluate the effect of the release of 24 h BOO on bladder function, we used non-invasive VSA to detect the voiding function changes. In the male mice, VSA revealed a decreased average spot area at 3 days post release compared with the control, while at 7 and 14 days post release, the average spot area returned to the same as the control. The female mice showed no changes in the average spot area compared with the control (Figures 2A,B). The average voided volume at 3 days post release was significant reduced in the male mice compared with the females (Figure 2B).

We analyzed 148 voids of all the control mice, of which around 80% (117/148) were under 30 μl and around 10% (14/148) were above 100 μl in the control mice (Figure 2C). We then chose 30 μl and 100 μl as the cutoff to analyze the changes of individual void in each group. The spot counts represented the void frequency. The male appeared to have an obvious increase in the void counts at 3 days post release, which were mostly due to voids less than 30 μl. However, this tendency did not reach statistical significance. The void count in different urine volumes did not change in the female mice through the observation (Figure 2D).

The total spot areas, which represented the total void volume in the 2 h, were not significantly changed through the observation period in either the male or the female mice (Figure 2E).

Taken together, these data indicate that release of BOO affected the bladder voiding function in male mice at 3 days, with decreased urine volume per void and a tendency of increased frequency in small volume voiding. No significant changes in frequency and urine volume per void were observed in the female. The urine production did not change during the release period.

### Assessment of Renal Function After the Release of Bladder Outlet Obstruction

**FIGURE 3:**
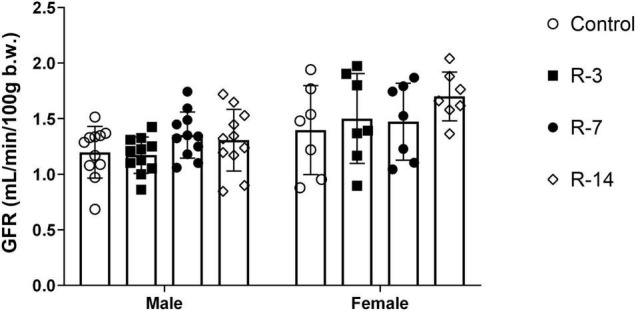
Renal function after release of 24 h total bladder outlet obstruction. The transcutaneous glomerular filtration rate (tGFR) of the mice were constant in both the male and female mice, indicating that renal function was not affected by the 24 h obstruction followed by release. (*n* = 11 in each group in the male mice and *n* = 7 in each group in the female mice). Data are presented as means ± SD.

To elucidate whether the renal function was affected after BOO and subsequent release, we performed tGFR to assess the glomerular filtration rate. Both genders showed no change in tGFR throughout the observation period (Figure 3). Thus, these data indicate that 24 h total BOO following by release did not affect the renal function.

### Changes of Col-1, Col-3 and Fibronectin in Response to the Release of Bladder Outlet Obstruction

**FIGURE 4:**
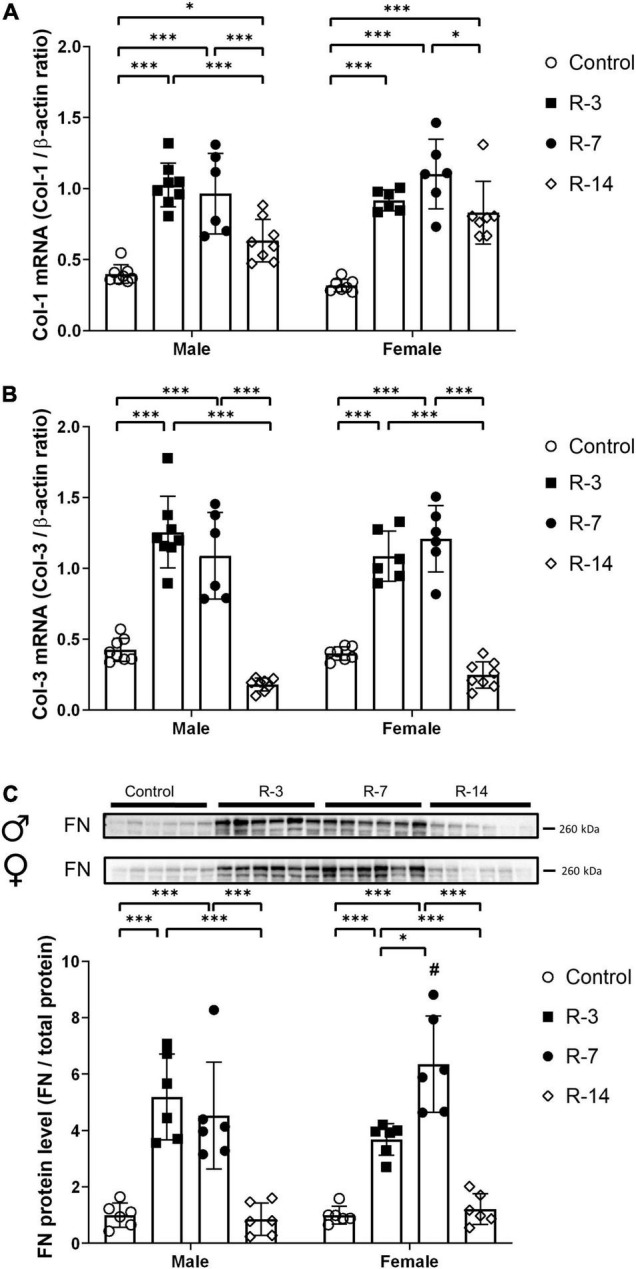
Changes of collagen I, collagen III, and FN expression during 14 days after release of 24 h total bladder outlet obstruction. **(A)** Col-1 mRNA expression was significantly increased at 3, 7, and 14 days post release in both genders. **(B)** Col-3 mRNA expression was significantly increased at 3 and 7 days post release, and it was back to control level again at 14 days post release. Relative mRNA expression was calculated using the reference gene β-actin (*n* = 8, 8, 6, and 8 in the male control, R-3, R-7, and R-14 group, respectively; *n* = 7, 6, 6, and 8 in the female control, R-3, R-7, and R-14 group, respectively). **(C)** Representative Western blots of FN showing increased FN protein levels at 3 and 7 days post release in both genders. At 14 days post release, FN protein levels were back to control levels. FN protein levels were lower in the male R-14 than in the female R-14 group. Quantification of protein expression relative was normalized by total protein (*n* = 6 in all groups). Male, ♀ Female. Data are presented as means ± SD. *P < 0.05 and ^***^P < 0.01. # *P* < 0.05 compared between genders with the corresponding group.

Next, we evaluated the changes in ECM markers, including collagen and FN. As shown in Figure 4, gene expression of Col-1 and Col-3 was significantly increased at 3 and 7 days post release in both male and female mice (Figures 4A,B). At 14 days post release, the increased expression of Col-1 mRNA was still present in both genders, while Col-3 mRNA expression was similar to the control levels. There was no significant difference of Col-1 and Col-3 gene expression between the male and the female mice. The FN protein level was significantly upregulated in both genders at 3 and 7 days post release, while at 14 days post release, FN was comparable to the control levels (Figure 4C). Compared with the female mice, the male mice showed a lower FN protein level at 7 days post release (Figure 4C). These findings suggested that both genders had an early increase in ECM markers, which decreased over time after the release of 24 h BOO.

### Changes of TGF-β and BMP-7 in Response to the Release of Bladder Outlet Obstruction

**FIGURE 5:**
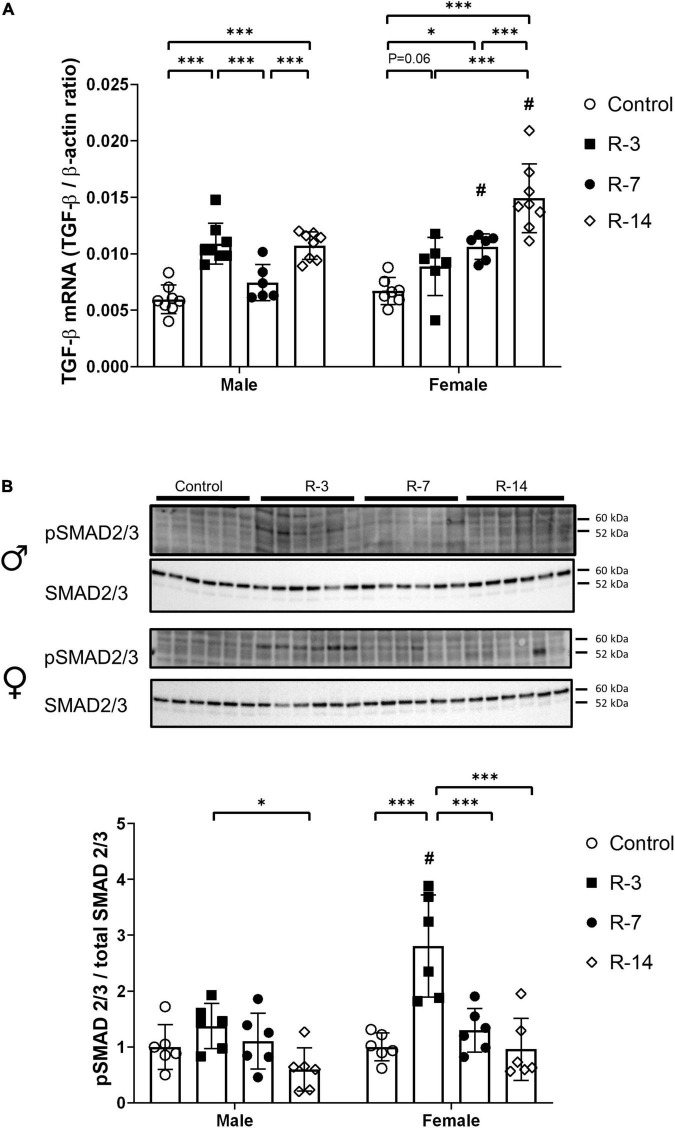
Activation of the TGF-β/SMAD2/3 signaling after release of 24 h total bladder outlet obstruction. **(A)** TGF-β mRNA expression was increased in male mice at 3 and 14 days post release, with a drop to control levels at 7 days post release. In female mice, TGF-β mRNA expression was significant increased at 7 and 14 days post release. The male mice showed a lower TGF-β mRNA expression in the R-7 and R-14 groups compared with the female R-7 and R-14 groups, respectively. Relative mRNA expression was calculated using the reference gene β-actin (*n* = 8, 8, 6, and 8 in the male control, R-3, R-7, and R-14 group, respectively; *n* = 7, 6, 6, and 8 in the female control, R-3, R-7, and R-14 group, respectively). **(B)** Representative Western blots related to SMAD signaling showing no activation of SMAD2/3 during the observation period in the male mice. Increased phosphorylation of SMAD2/3 were observed in the female mice at 3 days post release, then back to control level at 7 and 14 days post release. The pSMAD 2/3 of female R-3 group was higher than the male R-3 group. pSMAD 2/3 was normalized by total SMAD2/3. Quantification of protein expression relative was normalized by total protein (*n* = 6 in all groups). Male, ♀ Female. Data are presented as means ± SD. *P < 0.05 and ^***^P < 0.01. # *P* < 0.05 compared between genders with the corresponding group.

Next, we explored the impact on the TGF-β and BMP-7 signaling pathways, which play an important role in the regulation of fibrogenesis. In male mice, gene expression of TGF-β was significantly increased at 3 and 14 days post release compared with the control mice, while at 7 days post release, there was no change related to control mice. However, in the female mice, TGF-β expression was increased at 7 and 14 days post release compared with that of control mice, while the increase at 3 days post release showed borderline statistical significance (*P* = 0.06) (Figure 5A). Compared with the corresponding female mice, the male mice showed decreased TGF-β mRNA expression at 7 and 14 days post release (Figure 5A). Furthermore, we studied the activation of SMAD2/3 by phosphorylation, which is a key downstream protein element in TGF-β signaling. Phosphorylated SMAD2/3 measurements showed no significant changes in the male mice during the observation period compared with the control (Figure 5B). However, in the female mice, the phosphorylated SMAD2/3 was significantly upregulated at 3 days post release, while at 7 and 14 days post release, there was no change compared with that of control mice. The female mice showed a higher phosphorylated SMAD2/3 protein level at 3 days post release compared with that of the male mice (Figure 5B).

**FIGURE 6:**
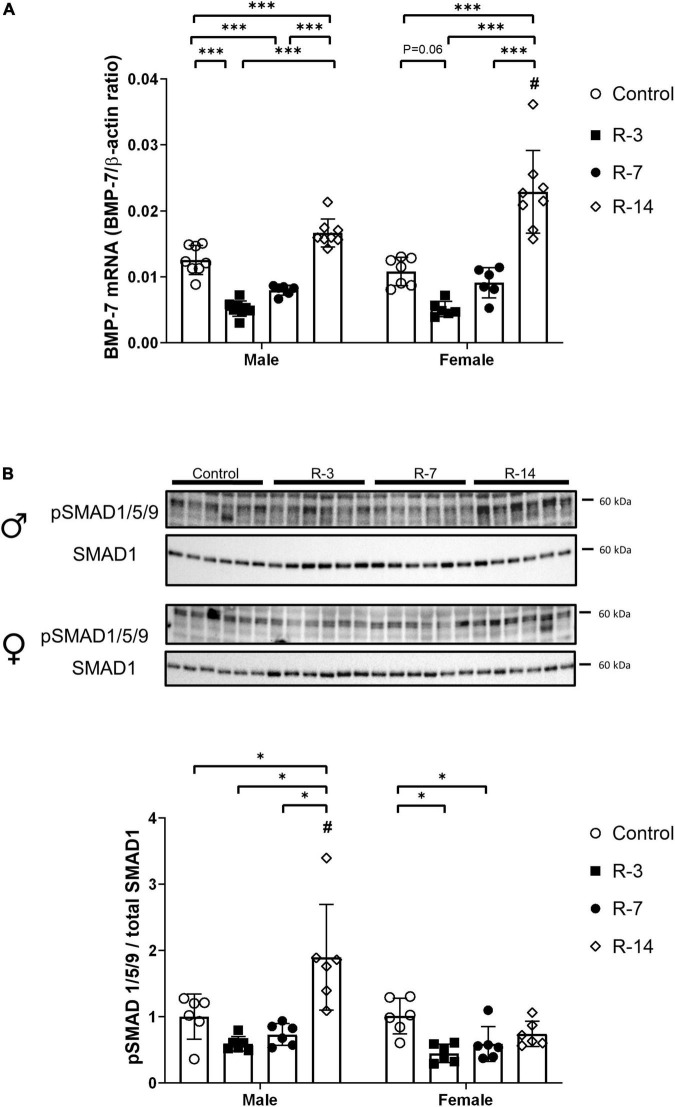
Activation of the BMP-7/SMAD1/5/9 signaling after release of 24 h total bladder outlet obstruction. **(A)** BMP-7 mRNA expression was decreased at 3 and 7 days post release, followed by increased levels at 14 days post release. In female mice, BMP-7 mRNA expression showed a tendency to decrease at 3 days post release, while at 14 days post release increased BMP-7 expression was observed. The BMP-7 mRNA expression in the male R-14 group was lower than that in the female R-14 group. Relative mRNA expression was calculated using the reference gene β-actin (*n* = 8, 8, 6, and 8 in the male control, R-3, R-7, and R-14 group, respectively; *n* = 7, 6, 6, and 8 in the female control, R-3, R-7, and R-14 group, respectively). **(B)** Representative Western blots related to SMAD signaling showing increased phosphorylation of SMAD1/5/9 in the male mice at 14 days post release. The pSMAD1/5/9 protein level in the male R-14 group was higher than that in the female R-14 group. Decreased phosphorylation of SMAD1/5/9 was found at 3 and 7 days post release in the female mice. pSMAD1/5/9 was normalized by total SMAD1. Quantification of protein expression relative was normalized by total protein (*n* = 6 in all groups). Male, ♀ Female. Data are presented as means ± SD. *P < 0.05 and ^***^P < 0.01. # *P* < 0.05 compared between genders with the corresponding group.

BMP-7 mRNA expression was significantly decreased in male mice both at 3 and 7 days post release compared with that of control mice. However, at 14 days post release, BMP-7 expression was increased compared with the control (Figure 6A). In the female mice, a decreased tendency of BMP-7 expression was observed at 3 days post release, but it did not reach significance (*P* = 0.06), while at 14 days post release, an increased BMP-7 expression was observed (Figure 6A). At 14 days post release, the male mice showed lower BMP-7 mRNA levels compared with the female (Figure 6A).

Then, we studied the activation of SMAD1/5/9, which is the classical downstream signaling pathway of BMP-7. Phosphorylated SMAD1/5/9 was significantly increased in the male mice at 14 days post release compared with the control, which was consistent with the increased BMP-7 expression. However, in the female mice, decreased phosphorylated SMAD1/5/9 was observed at 3 and 7 days post release, while at 14 days post release, there was no change compared with that of the control (Figure 6B). The male mice displayed a higher phosphorylated SMAD1/5/9 at 14 days post release compared with that of the female mice.

Collectively, these data indicate that TGF-β and BMP-7 signaling pathways played a role in the altered bladder wall changes during the bladder recovery in both male and female mice.

## Discussion

This study explored the recovery process of murine bladders after 24 h BOO followed by release for 3, 7, and 14 days, with reference to molecular events, histological, and functional changes of the bladder. Our results showed that after the release of obstruction, the bladder developed thickened walls with increased collagen deposition and upregulated fibrosis-related signaling pathways. When comparing between genders, the data showed that male mice responded to the 24 h BOO and release with early thickened bladder walls and significantly decreased urine volume per void, while these changes were either delayed or undetectable in the female mice. However, profibrotic molecular changes, including FN and TGF-β expression, seem to be higher at later time points in the female mice compared with those of male mice. The finding from this study suggested that in the male mice, the damage of 24 h BOO may induce an instant response in the bladder wall, which started to phase out at 14 days post release, indicating a compensated bladder repair process. However, the female mice responded more gradually in the beginning, which was delayed, but ongoing, and built up at 14 days post release, suggesting a decompensated repair and extending remodeling process in the female mice.

Bladder fibrosis is the end result from various bladder pathological conditions and characterized by thickened bladder walls and increased collagen deposition. Bladder wall thickness has been shown to be an accurate predictor in diagnosing BOO in men (16). Increased bladder wall thickening after partial BOO was reported in male (17) and female animals (18), while this is the first study of the bladder morphological and functional parameters in both genders. The male animals showed a thickened bladder wall through the release period, while in the female animals, the bladder wall was not thickened until 7 days and 14 days post release. This gender difference in morphological repair response calls for further studies.

Short-term urinary retention for 60 min was reported to develop fibrosis in the suburothelium layer in rats (19). In this study, collagen deposition was evident in the suburothelial layer after the release of 24 h BOO, with additional increased deposition in the smooth muscle layer in both genders at 7 and 14 days post release. Collagen I and III constitute the dominant elements of the bladder ECM, and normal compliance requires a balance between both collagen subtypes (20). Excessive or overabundance of collagen III and its localization in the smooth muscle layer are noteworthy to contribute to reduced compliance in bladder fibrosis (21, 22).

Bladder outlet obstruction can lead to pathophysiological bladder dysfunction in form of urinary urgency, which is the clinical hallmark for an overactive bladder (OAB) (19). In this study, besides the morphological changes, the alterations in the voiding pattern were an increased voiding frequency at 3 days post release in the males accompanied by small volume voids. The transiently increased frequency is a typical manifestation of OAB seen with BOO (19), and restoration of bladder function may occur after BOO relief as occurred at 7 and 14 days post release. This recovery of bladder function was not parallel to the histological alterations seen at the same time points and suggest that the anatomical histological changes may persist for a longer period from functional recovery.

By measuring the tGFR, we evaluated whether the renal function is affected by 24 h BOO followed by release. Decompression of severe obstructed bladder causes postobstructive diuresis, which is mostly the consequence of reduced renal function (23). The urine output recorded during the 2 h VSA showed no difference through the 14-day observation. Our findings in the tGFR did not support the hypothesis that renal function is affected by the releasing of 24 h BOO.

Transforming growth factor-β is a multifunctional growth factor, pathologically increasing proliferation by increasing ECM production. This study presented upregulated TGF-β through the obstruction release period in both genders. We have previously demonstrated that 24 h infravesical obstruction induced an immediate upregulation of TGF-β (6). In this study, except for a drop to the control level at 7 days post release in the male mice, we observed an elevated TGF-β level indicating bladder remodeling after the release of obstruction. Lin et al. (24) demonstrated a higher plasma TGF-β level in rats at 4 weeks after the release of partial bladder obstruction. Along with the persistent bladder wall thickening and collagen deposition, the continuing activated TGF-β further underlined its key role in the bladder remodeling during the obstruction release. The lower TGF-β mRNA expression at 7 days post release in the male mice was not found in the female mice. We cannot explain this unexpected finding, and the exact cause required further studies.

Bone morphogenic protein has shown its protective role in counteracting TGF-β activity during fibrosis by downregulating collagen expression and enhancing ECM degradation (25, 26). We have previously shown downregulation of BMP-7 after 24 h bladder obstruction (6). In this study, we detected an initially decreased expression of BMP-7 at 3 and 7 days post release, followed by increased expression at 14 days post release. The inverse changes of TGF-β and BMP-7 at 3 and 14 days together with the activated BMP-7-SMAD1/5/9 signaling at 14 days post release show an ongoing repair process, which occurs slowly after relief of the obstruction.

To have a comprehensive depiction of the bladder repair process, we included both male and female mice to evaluate whether they manifested differently. In this study, the bladder wall thickening was shown in both genders; however, it was first observed at 7 days post release in the female mice. The male mice showed increased bladder wall thickness at 3, 7, and 14 days post release, along with the small volume voids only observed in the male mice. The Sirius Red staining revealed a similar collagen fraction in the smooth muscle cell layer after the release of BOO in both genders. The differences in ECM markers were less pronounced between genders. However, FN and TGF-β expression was found to be slightly increased in the female mice compared with those in male mice at 7 and 14 days post release, suggesting more progressive fibrogenesis at the later stage in the female mice. Our previous study found that 24 h BOO developed a thin bladder wall with increased bladder weight in the female mice but not the male mice (6). Taken together, we speculate that the male mice might compensate more rapidly to the injury of the obstruction with thickened bladder wall, which phased out when 24 h obstruction is released, whereas, the female mice might develop bladder decompensation with large capacity and thin bladder wall, which could lead to an ongoing fibrotic response. Future studies of extended observation and bladder capacity evaluation are obligated.

We recognized limitations that could impact our gender differences in our bladder analyses. The application of total BOO in this study excluded the possible role of anatomical factors, e.g., the different urethral structures between genders that may mitigate the influence of BOO. Other potential factors, including sex hormones, could play a role. Sex hormone receptors, including those of estrogen, progesterone, and androgen, are located throughout the bladder and urethra in both genders (9), suggesting a potential relationship between hormones and the lower urinary tract function. A study using partial BOO mice showed that supplement of testosterone to castrated male mice mediated fibrosis in the bladder wall (17), indicating that testosterone aggravate fibrotic remodeling in the damage bladder in a long-term partial BOO model. However, the exact reasons are unknown. The role of sex hormones in both genders is warranted for future studies.

We used total obstruction to avert the concerns of the high variability in the obstruction degree, which was frequently encountered in the partial BOO model (5). The total obstruction was therefore applied to focus on the consistent bladder injury and the following repair. To investigate the potential treatment of bladder fibrosis, adequate models with high reproducibility are in great demand. We demonstrated a standardized total obstruction, which induced significant bladder wall thickening and altered collagen deposition, together with fibrogenesis-related molecular events during the repair process. In spite of the significant changes in the bladder remodeling, kidney function and urine production were not impaired, indicating that this model can be used to study isolated processes in the bladder. Thus, we recommend this animal model as a useful model for future bladder fibrosis studies to identify potential intervention methods.

Finally, we acknowledge that a time-matched sham-operated group could have provided a better control in order to show the isolated impact of obstruction and relief. Second, there is no bladder dry weight to exclude the effect of edema on bladder wall thickening. Third, the loss of nerve fibers and innervation of bladder was not evaluated, which might play an important role in bladder dysfunction after obstruction. Therefore, to improve the value of these results, future studies are necessary including measurement of bladder dry weight, nerve density, and blood testosterone/estrogen level.

## Conclusion

The release of 24 h BOO initiated a bladder remodeling process concerning voiding function and a series of fibrosis-related molecular events. The animal model enables a wide range of experiments to study bladder remodeling. The potential role of sex hormones in bladder repair based on our findings of gender differences warrants further study. Identification and characterization of the recovery process help future refine intervention strategies for bladder fibrosis.

## Data Availability Statement

The original contributions presented in the study are included in the article/supplementary material, further inquiries can be directed to the corresponding author.

## Ethics Statement

The animal study was reviewed and approved by Animal Experiments Inspectorate, the Danish Ministry of Food, Agriculture and Fisheries (Approval number: 2017-15-0201-01182).

## Author Contributions

YL, PA, JD, LO, and RN: conceptualization. YL, SM, IA, and RN: carry out the experiments and analysis the data. YL and RN: writing – original draft. YL, SM, IA, PA, JD, LO, and RN: writing – review and editing. All authors approved the final version of the manuscript.

## Conflict of Interest

The authors declare that the research was conducted in the absence of any commercial or financial relationships that could be construed as a potential conflict of interest.

## Publisher’s Note

All claims expressed in this article are solely those of the authors and do not necessarily represent those of their affiliated organizations, or those of the publisher, the editors and the reviewers. Any product that may be evaluated in this article, or claim that may be made by its manufacturer, is not guaranteed or endorsed by the publisher.
